# Supplementary material for: Peer support workers in co-production and co-creation in public mental health and addiction services: Protocol for a scoping review
Source: PLoS One. 2021 Mar 15;16(3):e0248558. doi: 10.1371/journal.pone.0248558 (PMC7959669; doi:10.1371/journal.pone.0248558)
Supplement: Attachment — Submitted filename: Response to reviewers.docx [file pone.0248558.s001.docx]

**Rebuttal Letter: Response to Reviewers PLOS ONE**

Manuscript title:

Peer support workers in co-production and co-creation in public mental health and addiction services: Protocol for a scoping review

Dear PLOS ONE Academic Editor and Reviewers,

Thank you for your very useful comments which provide very valuable insights to improve our current manuscript quality and coherency. Each of the statements given is well analyzed and provided with necessary explanation, correction and response to support the manuscript's points. Hence, below we addressed your comments and responses. First, we will address the reviewer´s response to Questions 1 and 4, then we will address each reviewers specific comments:

**Responses to question 1 and 4:**

1. **Does the manuscript provide a rationale for the proposed study, with a clearly identified and justified research questions?**

The research question outlined is expected to address a valid academic problem or topic and contribute to the base of the knowledge in the field.

*Reviewer 2# Partly.*

We have revised the research question *to: How are peer support workers involved in co-production and co-creation in public mental health and addiction services and, what are the described outcomes?*

This has also led to a refinement of the title to “Peer support workers in co-production and co-creation in public mental health and addiction services: Protocol for a scoping review”

4. **Have the authors described where all data underlying the findings will be made available when the study is complete?**

*Reviewer 1: No.*

Author comments: This information has been updated and answered in the comments no 6, below. The protocol was registered in Protokols.io: 2021.02.11. The study protocol and, all available data, will be made public when the study are conducted and published.

The study protocol and, all available data, will be made public when the study finishes.

**Comments and responses to each reviewer’s feedback:**

**Reviewer 1:**

Thank you for your thorough feedback. Your comments made us able to deliver an enhanced and better manuscript. To respond clearly to all comments, they are numbered so that it is easier to follow:

1) ***Reason for "major review" selection: Include a discussion at the end for the potential impact of the scoping review on the field.***

**Authors response:** A discussion at the end of the research protocol is included. The discussion part is characterized by the fact that it is a research protocol. This implies that we have pointed to the reasons for doing this study and what we expect to find based on our research question and why this knowledge will be of importance for policy, practice and research.

2) **Remove info on how data will be collected in "the aim of study" section. This more appropriately belongs in "methods".**

**Authors response:** Information on how the data will be collected are removed from "Aims of scoping review" to "Method".

3) **Include the visual you discussed about the study selection process, inclusion and exclusion.**

**Response:** A visualization of the study selection process based on PRISMA-Scr is added.

4) **PSW or PSWs? In some sentences, plural seems to fit better with certain sentences and grammar.**

**Authors response:** We have revised and consequently use PSW's. This fits better with grammar, as you suggest. However, the plural form works best because we address PSW's as employees in the services and not the individual employee.

5) **All acronyms are spelled out the first time when they are used. For instance, (United Nations, line #).**

**Authors response:** Thank you for making us aware of this mistake. Due to the refinement of this section, this sentence is no longer part of the introduction.

6) **Need to describe where data will be made available when study is complete.**

**Authors response:** The method section is updated with this information: The protocol was registered in Protokols.io: 2021.02.11. The study protocol and, all available data, will be made public when the study are conducted and published.

The study protocol and, all available data, will be made public when the study finishes.

7) **Include clear dates of articles included in study. Also define what inception is. Is it the start of journal, etc.?**

**Authors response:** This information is updated in the Method section, under the subheading, “study selection”: The search in databases will be from the inception of each database chosen until 04.01.2021.

**Comments & Response for Reviewer 2:**

Thank you for your thorough and useful feedback. Your comments made us able to revise and enhance the quality and arguments in our manuscript. Here, we will address your feedback:

**Comment 1:** Abstract is not traditional written, which is fine. I see authors report background and method. Final sentence in abstract is research question. I believe abstract will be improved if authors add a one or two sentences regarding any expected results and how these results will inform field.

**Authors response:** We have tried to improve the abstract, regarding sentences on expected results and how these results will inform field are included at the end. The text has been refined, and the abstract is more traditionally written.

**Comment 2:** Although authors defined co-creation and co-production. It is still difficult to understand the difference between co-creation and co-production. The distinction between co-creation and co-production could be explained with an example.

**Authors response:** The difference between the two concepts is now more precise defined in the text. First, in the abstract and then, this has been explained more thoroughly in the introduction, with examples.

We will address comment 3 and 4 together:

**Comment 3:** Second paragraph in introduction has some parts that could be moved to purpose of study. For example, line 93 and rest is about purpose of study. Interestingly, in "Aim of the Study" section authors talk about background again. I suggest authors to move all background information into introduction section. I also suggest authors have a *brief and concise purpose of study.*

**Comment 4:** There are too many questions in introduction. It is very hard to overall understand introduction due to all questions and how introduction is organized. I suggest authors to be more concise with purpose section and improve background section.

**Authors responses to comments 3 and 4:** The introduction section has been substantially modified. There are no questions in the introduction. The introduction starts with a background section where the research status and theoretical and practical assumptions for this study are accounted for. The reason to split between the concept, co-production and co-creation is explained, as the rationale for doing this study and in choosing this approach. The last section in the introduction describes the aim of this scoping review. We argue that PSW's different involvement in the interactive processes can influence their ability to contribute to innovative changes and that we need to pay attention to their involvement in co-creation processes because they have an innovative dimension not found in co-production.

**Comment 5:** I wonder whether this following sentence should move to Method: "The data will be collected based on Arkzey and O'Malley's framework (10): 1) identify the research questions, 2) search for relevant studies, 3) select studies, 4) chart the data, and 5) collate, summarize and report studies."

**Authors response:** This sentence has been moved to the Method section.

**Comment 6:** What year period authors aim to conduct this study? Any time restriction such as from 2010 to 2020.

**Authors response:** This is corrected in the Method section to be: From the inception of each included database until January 04.01.2021.

**Comment 7:** In any disagreement, what is the plan? I see the authors reported that they would have a discussion on disagreement to resolve it. What will be the focus of discussion? What is further step if there is no resolution? For example, do authors plan to bring a third reviewer to help resolution.

**Authors response:** This is corrected to be: The focus of discussion will be if articles clearly describe interactive processes, and if PSW's involvement in the process is clearly explained. If needed, disagreements will further be discussed and solved with the PhD candidates extended supervisory team, as this review article is part of a PhD project.

**Comment 8:** I see that editorials, reports, and opinion papers will be removed. Later, in "Information on the Following Categories....", item 4 has opinion papers and others.

**Authors response:** Thank you for making us aware of this mistake. We will not keep information about opinion papers. This has been deleted from the sentence.
